# Delimiting Species Using Single-Locus Data and the Generalized Mixed Yule Coalescent Approach: A Revised Method and Evaluation on Simulated Data Sets

**DOI:** 10.1093/sysbio/syt033

**Published:** 2013-06-14

**Authors:** Tomochika Fujisawa, Timothy G. Barraclough

**Affiliations:** ^1^Department of Life Sciences, Imperial College London, Silwood Park Campus, Ascot, Berkshire SL5 7PY, UK; and ^2^Department of Entomology, Natural History Museum, London SW7 5BD, UK

## Abstract

DNA barcoding-type studies assemble single-locus data from large samples of individuals and species, and have provided new kinds of data for evolutionary surveys of diversity. An important goal of many such studies is to delimit evolutionarily significant species units, especially in biodiversity surveys from environmental DNA samples. The Generalized Mixed Yule Coalescent (GMYC) method is a likelihood method for delimiting species by fitting within- and between-species branching models to reconstructed gene trees. Although the method has been widely used, it has not previously been described in detail or evaluated fully against simulations of alternative scenarios of true patterns of population variation and divergence between species. Here, we present important reformulations to the GMYC method as originally specified, and demonstrate its robustness to a range of departures from its simplifying assumptions. The main factor affecting the accuracy of delimitation is the mean population size of species relative to divergence times between them. Other departures from the model assumptions, such as varying population sizes among species, alternative scenarios for speciation and extinction, and population growth or subdivision within species, have relatively smaller effects. Our simulations demonstrate that support measures derived from the likelihood function provide a robust indication of when the model performs well and when it leads to inaccurate delimitations. Finally, the so-called single-threshold version of the method outperforms the multiple-threshold version of the method on simulated data: we argue that this might represent a fundamental limit due to the nature of evidence used to delimit species in this approach. Together with other studies comparing its performance relative to other methods, our findings support the robustness of GMYC as a tool for delimiting species when only single-locus information is available. [Clusters; coalescent; DNA; genealogical; neutral; speciation; species.]

A fundamental pattern of nature is that organisms diversify into more or less discrete entities that we call species. The term “species” is used to encompass many facets of the pattern of diversity and its underlying causes (Hey 2001; Coyne and Orr 2004; De Queiroz 2007; Shaffer and Thomson 2007). However, in general evolutionary terms (e.g., as encapsulated by the general lineage concept of species, De Queiroz 2007), species are groups of organisms that evolve independently from other such groups (because of barriers to the spread of genes from one species to another), which therefore diverge into discrete units of morphological and genetic variation apparent from surveys of higher clades. Increasingly, studies on the nature and origins of species use empirical tools to delimit evolutionarily significant taxa based on measurable quantities, for example, reproductive isolation (Coyne and Orr 1998; Knowles and Carstens 2007), ecological divergence (Fontaneto et al. 2007; Leaché et al. 2009), and other traits (Feulner et al. 2007), rather than relying on qualitative taxonomic methods (see discussion by Sites and Marshall 2003).

Gene trees provide a valuable source of information for inferring the pattern and processes of diversification. Until recently, studies of diversification were constrained by a trade-off in numbers of loci, numbers of individuals per species, and numbers of species that could be sampled, leading to either data sets of many loci in a few species or a few loci for many species. Population genetic and phylogeographic studies investigated population history and gene flow in related sister species or species complexes (Templeton 2001; Avise 2009). These studies typically sampled large numbers of individuals per species and often multiple molecular markers (Koufopanou et al. 1997), yet because of the level of sampling needed to infer population processes, by necessity a given study usually focused on few species. In contrast, phylogenetic studies reconstructed speciation events leading to extant species and inferred macroevolutionary processes at broad taxonomic and geographical scales (Barraclough and Nee 2001). However, because phylogenetic studies require a large sample of species for a large clade, by necessity these studies, until recently (Carstens and Dewey 2010; Camargo et al. 2012), mostly sampled only one exemplar per taxonomic species. Theory to analyze population versus phylogenetic data also developed separately.

It is now feasible to sample multiple individuals from most species across a wider clade. For example, DNA barcoding projects perform molecular inventories of large samples of species and multiple individuals within species (Hebert et al. 2003; Meyer and Paulay 2005; Monaghan et al. 2009). Similarly, environmental samples of unculturable organisms, such as bacteria and microbial eukaryotes, can be sequenced from marine and terrestrial ecosystems (Acinas et al. 2004). These data offer the potential to delimit evolutionarily significant units of diversity at broad taxonomic scales. However, at present, large-scale biodiversity surveys still mostly rely on single loci, such as cytochrome oxidase I for animal barcodes or 16S rDNA for bacterial diversity surveys (note that plant DNA barcoding uses multiple loci but often multiple linked loci on the plastid genome, which equates to a single locus in terms of genealogical information, see CBOL Plant Working Group 2009). Despite advances in genomic technology, it remains difficult to sample multiple variable and orthologous nuclear markers at equivalent taxonomic breadth and depth (but see Brito and Edwards 2009). In addition, for unculturable organisms, sampling multiple markers from individuals (as opposed to pooled environmental samples sensu Venter et al. 2004) is challenging (Barraclough et al. 2009). Evolutionary inference from single-locus data has limitations, including lower power for detecting independent evolution compared with multilocus approaches (Knowles and Carstens 2007; Dupuis et al. 2012), the potential discordance between gene trees and species trees (Hailer et al. 2012), and the lack of information on adaptive divergence (Will and Rubinoff 2004). Nonetheless, single-locus data do provide a genetic record of evolutionary histories (Avise 2009) and, therefore, provide useful information for surveying evolutionary patterns of diversity across broad scales (Monaghan et al. 2009).

Several methods have been proposed that are suitable for delimiting species from single-locus data. The simplest approach is to define species based on a percentage cut-off rule, such as the 1% or 3% rule used to delimit bacterial species from 16S rDNA sequences (Schloss and Handelsman 2006) or cytochrome oxidase I for insect species (Brower 1994). This method suffers from a weak connection to evolutionary theory, from variation in typical levels of intraspecific and interspecific variation among clades, and from substitution rate variation among lineages (Barraclough et al. 2009). Although pairwise distance thresholds might often work well in practise (Tang et al. 2012), evolutionary methods are needed to validate their use. Also, an underlying evolutionary model is needed to assign uncertainty in the resulting delimitation and to compare alternative evolutionary hypotheses statistically, such as whether a clade has diversified into species or not (e.g., Fontaneto et al. 2007).

Evolutionary methods have focused on detecting genetic signatures indicative of independent evolution, such as evidence of fixed differences (Hey 1991; Davis and Nixon 1992) or reciprocal monophyly (Wiens and Penkrot 2002) between population samples. However, these approaches require a priori hypotheses of putative species groupings, based on traditional taxonomy, morphospecies, or population samples, against which criteria such as monophyly can then be assessed. This can introduce biases into delimitation (e.g., cryptic sympatric species would not be delimited because there would be no independent data for judging monophyly or fixed differences) and the additional information required is not available in extreme cases, such as delimiting bacterial species from a single environmental sample solely from DNA sequence data (Acinas et al. 2004; Venter et al. 2004). In more recent studies, Bayesian methods to delimit species using multilocus sequence without a priori species boundaries have been proposed (O'Meara 2010; Yang and Rannala 2010). These approaches can handle uncertainty of delimitation and utilize multilocus data. However, they are currently too computationally intensive to apply to large samples.

The Generalized Mixed Yule Coalescent (GMYC) method, devised by T.G.B. and first introduced in Pons et al. (2006) and Fontaneto et al. (2007), is one method designed to delimit independently evolving species using single-locus data. By “independent evolution,” we mean that new mutations arising in one species cannot spread readily into another species (Templeton 1989; Barraclough et al. 2003; De Queiroz 2007). The GMYC method relies on the prediction that independent evolution leads to the appearance of distinct genetic clusters, separated by longer internal branches (Barraclough et al. 2003; Acinas et al. 2004). It delimits such genetic clusters by optimizing the set of nodes that define the transitions between inter-and intra-specific processes. Optimization proceeds by finding the maximum likelihood (ML) solution for a model that combines diversification between species (based on a Yule model, Nee et al. 1994) and genealogical branching within species (based on a neutral coalescent, Hudson 1990). Because it does not rely on additional evidence to formulate hypotheses of putative species, the method can be applied in cases lacking additional data with which to infer putative species limits. Other methods have been proposed based on similar predictions to those used by GMYC, and these often lead to similar outputs (e.g., the K/theta method of Birky et al. 2010). An advantage of GMYC is that the likelihood framework allows for statistical inference and hypothesis testing across the entire sampled clade. The relative performance of GMYC and alternative methods is compared elsewhere (Birky et al. 2010; Tang et al. 2012).

The method has been applied to single-locus data of many organisms (e.g., Fontaneto et al. 2007; Lahaye et al. 2008; Papadopoulou et al. 2008), and extensions of the algorithm have been proposed (Monaghan et al. 2009; Powell 2012). The method has not, however, previously been fully described or had its performance evaluated against simulated data under a wide range of conditions (although see Papadopoulou et al. 2008 for the effects of restricted dispersal and Esselstyn et al. 2012 for the effects of varying effective population sizes and speciation rates). Here, we present the algorithms used in detail and determine the power and error rates on data sets simulated under a wide range of conditions. In addition, we describe the statistical properties of the GMYC delimitation method, and make an adjustment in its formulation. The threshold times used to specify the location of nodes defining species are now correctly treated as a model constraint, rather than (incorrectly) as a parameter. This change does not affect the ML delimitation found by the method, but it does affect the power to reject the null model that all individuals belong to a single species cluster.

## Methods

### Assumptions

Assume that a single locus has been sequenced for a sample of individuals from a monophyletic clade. The sample is sufficient that multiple individuals have been sampled per species, should different species be present, and that most if not all species are sampled. Different sampling schemes will be considered below. Assume that the true genealogy of the locus is known (i.e., there are no reconstruction artefacts), that the locus is a neutral marker, and that there is no geographic substructure within each species. Departures from these assumptions will be discussed below. The goal is to determine whether the clade has diversified into independently evolving groups, namely species and, if so, to delimit those species.

The null model is that all individuals within the sample belong to a single species or population. The expected patterns for gene trees in a single population are well established. Sampled genes coalesce to a single common ancestor at a mean rate proportional to the effective population size, *N*_e_, in the case of a strict neutral coalescent (Hudson 1990; Rosenberg and Nordborg 2002). Coalescence occurs because population size is limited: the chance that each individual contributes offspring to subsequent generations depends on the contribution of the other individuals in the population.

**Figure 1:**
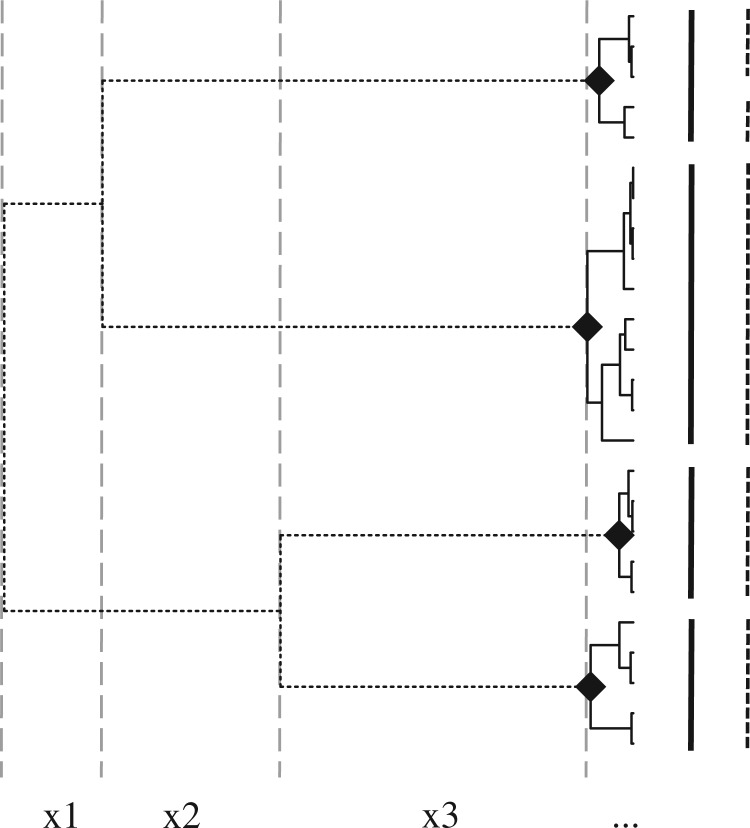
Schematic diagram showing how nodes are used to define species on a hypothetical gene tree. Black diamonds indicate the MRCA nodes that define species in a particular delimitation. Branches in dashed and solid lines represent speciation and coalescent branches, respectively. Bars next to the tips represent two alternative delimitations. Solid bars indicate the delimitation defined by the MRCA nodes shown on the tree. Dashed black bars indicate an alternative delimitation (i.e., splitting the top species into two) that could be assigned by the multiple-threshold method but not by the single-threshold method. Dashed gray vertical lines show the waiting times ×1, ×2, etc. that are used in the GMYC likelihood model.

Our alternative model is that the clade has diversified into separate species, each of which is considered as a single entity in the sense outlined for the null model. Gene copies will tend to coalesce back to a single common ancestor within each species. If the time to most recent common ancestor (*Tmrca*) within species is younger than the time since the species split from their nearest sisters, this will lead to a pattern of genetic clusters: clusters of closely related individual separated from other such clusters by longer internal branches (Fig. 1; Barraclough et al. 2003). Branching rates between clusters will reflect speciation events, as well as extinction and the degree of sampling of species entities (Nee et al. 1994; Barraclough and Herniou 2003). Branching within clusters will reflect the same population processes outlined for the null model but now occurring independently in separate species. However, if the *Tmrca* within species tends to be older than the time since each split from its nearest sister species, then clusters may not be observed and we may be unable to reject a null model of no species diversification. Note that our definition is similar to the general lineage concept of species although it differs in focusing on the genetic signatures that we use to delimit such groups (De Queiroz 2007).

The above scheme assumes two extreme cases: one unstructured population versus diversification into two or more populations, each of which is unstructured but fully isolated from the others. In reality, there will often be some degree of geographical structure within most species as well (Avise 2009). This could lead either to signatures intermediate between the two extreme cases or to hierarchical clustering, with population clusters apparent within species clusters (Lohse 2009; Papadopoulou et al. 2009). Expressions are available for scenarios departing from the assumptions of neutrality, constant population size, and unstructured populations (e.g., Charlesworth et al. 2003). For now, we ignore this complication and address how to detect significant signatures of diversification in the simple case. Note that this issue does not affect application of the method to delimit species within a single environmental sample, where geographical subdivision within species cannot occur.

### Likelihood Model

The GMYC approach extends existing likelihood methods for analyzing the timing of branching events in gene trees. The raw data for comparing different models are waiting times between successive branching events, *x_i_* (Fig. 1). Under the null model that the entire clade represents a single population (Hudson 1990), the likelihood of waiting time *i* under a neutral coalescent model with an effective population size of *N*_e_ and *n* lineages present is given by:
(1)
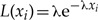

(2)
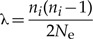

The alternative model is that the clade has diversified into *k* species, each of which is treated as an independent population with effective size, *N_j_,j* = 1 to *k*. The model splits the tree into two types of branching events. First, branching events within species are determined by *k* independent neutral coalescent processes (shown by the black branches on Fig. 1). Second, branching among species (shown by the dashed branches on Fig. 1), which we refer to as diversification, is treated as a stochastic birth–death process as developed for analyzing speciation and extinction on species-level phylogenetic trees (Nee et al. 1994; Morlon et al. 2011; Etienne et al. 2012). By convention, the simplest model is a Yule model that the number of species grows exponentially over time, with constant average birth rate, *λ*_spec_, and no extinction. The likelihood of waiting times between successive branching events among-species is:
(3)
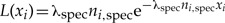

We modified the diversification process slightly to permit comparison of null and alternative models. By convention, phylogenetic branching models take a forward perspective, starting at the root and having *M*-2 speciation events, where *M* is the number of tips. Coalescent approaches take a reverse perspective, starting at the tips and having *M*-1 coalescent events. Hence, if we took a forward time approach for the diversification part of our mixed model we would have one less event than in the null model that the sample is drawn from a single population. To account for this, we adopt a reverse approach for the diversification branching process, that is, starting from the stem branches for each species, using equation (3) as the likelihood that the previous branching event occurred *x_i_* time units previously (cf. Hey 1992). A further complication is that stem branches leading to each species have a different probability distribution than either diversification or coalescent branches, because (in reverse time) each stem branch starts at a common ancestor node for an entity and ends at a diversification event. The simplest approximation, which we adopt, is to assume that these branches have the same probability distribution as the diversification branching process.

The final step is to calculate the likelihood of observed waiting times on the tree assuming a mixed model of coalescence within species and diversification among species. The combined series of events resulting from the combination of *k* independent Poisson processes follows a Poisson process with rate *b* equal to the sum of the rates of the separate processes (Cox and Miller 1965, p. 154). Hence, for a Yule diversification process and a set of *k* neutral coalescent processes within a given assignment of species, the likelihood of each waiting time is:
(4)
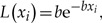

(5)
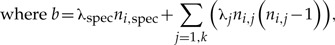

where *λ*_spec_ and *n*_spec_ are used to indicate the diversification process, and *n_i,j_* is the number of lineages in waiting interval *i* belonging to process *j*. Calculation of the relevant number of lineages for waiting times must take account of which lineages at any time point are taking part in which branching process (Fig. 1). The model is fitted by maximizing the sum of log likelihood of waiting times for both classes of branching events across the entire tree. Note that, in common with previous approaches for modeling diversification processes (Hey 1992), we do not model gene tree topology (cf. Rannala and Yang 2003), but instead model the distribution of waiting intervals between branching events.

### Generalized Model

The above equations make strict assumptions about the constant rate of branching in both population and diversification processes that seem unlikely to hold widely. One solution would be to compare a large array of specific population and diversification models using the Akaike Information Criterion (AIC, Rabosky and Lovette 2008). For example, a birth–death branching process for diversification could be added by substituting the equations from Nee et al. (1994). This exercise might be useful for specific hypotheses of prior interest, but it is unwieldy due to the vast number of possible models, many of which might be indistinguishable (Kubo and Iwasa 1995; Barraclough and Nee 2001). An alternative solution is to generalize the above equations to fit a range of qualitatively different models, without specifying the underlying processes. Nee et al. (1994) and Nee (2001) discussed transformations of waiting intervals that allow derived models to be treated as exponential processes with a single rate parameter. These accounts imply a simple general expression for the likelihood of waiting times of a single branching process:
(6)
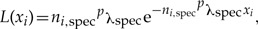

where *p* is a scaling parameter. A value of *p*= 1 indicates a constant per lineage branching rate, *p>* 1 indicates that the apparent branching rate accelerates toward the tips, and *p*< 1 indicates an apparent deceleration in rate toward the tips. For coalescent processes within entities, *n_i_* is substituted with *n_i_*(*n_i_* – 1).

The value of *p* is optimized during model fitting, allowing a range of qualitatively different models. Interpretation of *p* depends on which class of branching events are considered. Barraclough and Nee (2001) reviewed interpretations of changes in diversification rate on phylogenetic trees, which apply exactly to interpretation of *p*. For example, *p>* 1 indicates an apparent increase in diversification rate toward the present, as might be expected under a real increase in birth rate or under a model with a constant birth rate but adding a constant death rate (Nee et al. 1994). *p*< 1 represents an apparent decrease in diversification rate toward the present, consistent with niche-filling models, or incomplete sampling of species within the clade (Pybus and Harvey 2000; Nee 2001; Phillimore and Price 2008; Rabosky and Lovette 2008).

Similarly, *p* for a coalescent model will reflect processes affecting the timing of coalescent events within entities. *p*< 1 indicates a deficit of recent coalescent events, for example, if populations are growing in size or if the marker has experienced a selective sweep. *p>* 1 indicates an excess of recent coalescent events, for example, if populations were declining in size, if there was geographic subdivision within species or as a result of balancing selection on the marker. (Note that the description in Pons et al. 2006 of interpreting the effects of selection on coalescent *p* was incorrect.) Hence, by optimizing across possible values of *p* for both classes of branching events, our method is qualitatively robust to the details of the models. Note that the power transformation might not provide a linear approximation to particular process. However, it does allow smooth changes in branching rate over time to be fitted, and the fitted parameter values indicate which detailed models might be explored as possible explanations for the data.

Using the notation outlined earlier, the GMYC model is specified by substituting *b* in equation (5) with b^*^:
(7)
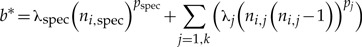

The ML estimate of *λ* for a given assignment of nodes is calculated using the Moran estimator of Nee (2001), namely the number of branching events divided by the total length of between-species branches. The estimator is modified to incorporate the scaling of branch lengths, that is, the number of diversification events divided by the sum of (*n*_*i*,spec_)^*p*_spec_^ *x_i_* across all waiting intervals. The equivalent expression but using (*n_i,j_*(*n_i,j_* – 1))^*p_j_*^ *x_i_* for the denominator is used for the coalescent processes. The scaling parameters are optimized using Nelder–Mead optimization as implemented in the “optim” function of R.

The simplest formulation is to assume that all the sampled entities have the same branching parameters, which we call the minimum model. In reality, different sampled entities might have different parameters, if their population sizes differ or they have experienced different demographic or selective histories. A maximum model would include separate *λ_j_* and *p_j_* for each entity. A more frugal approach is to allow for variation in parameter values among entities but only introducing a few additional parameters to the minimum model. We have tried fitting separate parameters for each cluster and results do not seem to greatly differ (see Pons et al. 2006) although future work could explore this further. For conciseness and simplicity we only consider the minimum model version here.

### Single-Threshold Approach to Delimiting Species

Delimitation with the GMYC approach is based on assigning branching events to two categories, speciation and coalescent within species. With the simplifying assumption that species are monophyletic, a set of most recent common ancestor (MRCA) nodes can be specified that determines the type of branching events (Fig. 1). Branches descending from the MRCA nodes are coalescent branches within the species, and clades defined by each MRCA node are species clusters. Because a given set of MRCA nodes specifies a form of the likelihood function, equation (7), and represents a unique testable hypothesis of memberships of putative species, each set of MRCAs can be treated as a candidate model of delimitation. The models are compared to obtain the best set of MRCA nodes within a ML scheme. Equations (6) and (7) are first optimized separately for each model then the likelihood values of the optimization results are compared. The set of MRCAs with the highest ML is selected as the best model of delimitation. This process is analogous to the two-step process of phylogenetic inference with ML, which is treated as model selection instead of parameter optimization (Yang et al. 1995; Yang 2006).

The challenge is that, even with the simplifying assumption of monophyly and a gene tree of modest size, there is an enormous number of possible candidate delimitation models (approximately 4.11×10^22^ models for a balanced tree with 128 tips, online Appendix 1). The simplest approach, proposed by Pons et al. (2006), is to assume that there is a threshold time, *T*, before which all nodes reflect diversification events and after which all nodes reflect coalescent events. This reduces the number of candidate models to equal the number of nodes in the tree. It does not assume that all species have the same *Tmrca*, which would be violated even in an equal population size model due to the expected variance in the *Tmrca*. Instead, it assumes that the most recent diversification event occurred before the oldest within-species coalescent event. If we assume for parsimony that all species have the same effective population size, this version of the GMYC model introduces two additional parameters compared to the null model: a diversification rate parameter, *λ*_spec_, and scaling parameter, *p*_spec_. The threshold time, *T*, which was treated as a parameter in Pons et al. (2006), is now correctly treated as a constraint of search space. The ML threshold, and hence assignment of species, is found by calculating the likelihood of the alternative model for all possible values of *T*. Multimodel comparison with the AIC can be used to assess the relative importance of alternative models and whether the ML alternative model is preferred over the null model of no such shift in branching process (Powell 2012). Note that with the reformulation of *T* as a constraint rather than a parameter, then the degrees of freedom for a log likelihood comparison of the null model with the single-threshold model is now 2, not 3 as proposed in Pons et al. (2006). The ML solution is unaltered with the new formulation, but the power is: any result using the former formulation with a *P*-value of 0.112 or less will be significant at 0.05 with the revised model.

### Multiple-Threshold Approach to Delimiting Species

The multiple-threshold approach, devised by TF and first described in Monaghan et al. (2009), relaxes the assumption of the single-threshold version that speciation events are older than all coalescent events in the gene tree. Instead, from a given starting assignment of MRCA nodes, it searches alternative models via an heuristic algorithm that iteratively splits and fuses existing species clusters (see fig. 1 in Monaghan et al. 2009). The algorithm keeps exploring additional sets until the last round of trials found no sets of MRCA with an improved likelihood on the previous ML set. We tried two versions of this process differing in the how the starting set of MRCA nodes is chosen. First, we started with arbitrary sets as described in Monaghan et al. (2009). In this case, optimization is often attracted to local optima, necessitating repeats with multiple starting sets, which greatly increases the run time. Second, we started with the set of MRCA found using the single-threshold method. To check the performance of these approaches we simulated 100 gene trees assuming a constant speciation rate and a population size of 10^5^ (as described further below) with four species and five tips per species; small enough to allow an exhaustive search of all delimitation models. These simulations showed that the multiple threshold starting from the best single-threshold solution more frequently finds the global optima and better solutions than the search starting from multiple arbitrary sets (Supplementary material at Dryad, doi: 10.5061/dryad.0hv88, Table S1, Fig. S1). We therefore use this approach.

In Monaghan et al. (2009), we treated additional thresholds needed to assign classes in the multiple-threshold algorithm as additional parameters, but here we redefine these as a constraint of search space rather than parameters, for the reasons described earlier. Therefore, the numbers of parameters are now identical in both the single-threshold version and the minimal model version of the multiple-threshold version. The two versions can no longer be compared by likelihood ratio tests, because they contain the same number of parameters. Instead, alternative assignments are considered together in a multimodel comparison framework.

### Uncertainty in Delimitation

We specify uncertainty in assignment using the AIC-based approach for multimodel comparison described by Powell (2012). Akaike weights and model-averaged estimates of GMYC parameters and their standard errors are defined in Powell (2012, equations 4–9). Also, the *α*% confidence set of candidate models can be obtained by adding the Akaike weights cumulatively starting from the minimum AIC model with increasing order until *α*% is attained (Burnham and Anderson 2002). The support value of a species cluster, which we call GMYC support, is given by the sum of the Akaike weights of models in which the MRCA node appears as follows.
(8)
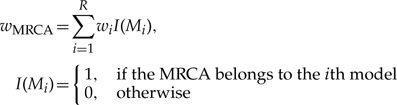

where *w_i_* is the Akaike weight of *i*th model, *M_i_* and *R* are the number of total model considered. Note that this represents support for the node among the alternative models of delimitation considered during model comparison. Although not all possible delimitation models can be compared, comparison of models specified by all possible single thresholds together with additional models searched with the multiple-threshold algorithm is a judicious approach.

### Simulations

To assess the properties of the method, genealogies were simulated under several scenarios of evolution and sampling schemes, which were expected to affect the performance of the method. In each following treatment except for A, species trees were first simulated under different diversification models, then gene genealogies within the species trees were simulated. One hundred replicated gene trees were simulated for each scenario. Species trees were simulated in Phylogen (Rambaut 2002) and gene trees in ms (Hudson 2002), using scripts in R to generate ms command files specifying phylogenetic history among species based on the simulated species tree. We modified ms to output branch lengths to seven decimal places instead of three to avoid the simulation of simultaneous coalescent events for the large samples being studied here.

(A) Null model: The null model was simulated assuming a neutral coalescent process in a single population and a sample of 150 individuals. Branch lengths in the resulting trees are in units of 4 *N*_e_ generations. Because the GMYC method relies on relative branch lengths rather than absolute branch lengths, we scaled branch lengths in the simulated trees to have a root age of 1.0 before running the GMYC analyses, to maintain consistent scale across the different simulations.
(B) Diversification (coalescence within a species tree): Gene trees were simulated assuming 30 independently evolving species. First, species trees were simulated under a constant birth (Yule) model without extinction. Species trees were scaled so that the root node age was 10 million generations, yielding a net per lineage speciation rate of approximately (log(30)-log(2))*/*10^7^ = 0.27, which falls within typical values (Barraclough and Vogler 2002). Then, gene trees within species trees were simulated assuming that five individuals were sampled from each entity. Numbers were chosen to match realistic yet conservative sample sizes (e.g., Pons et al. 2006). The effects of sampling more individuals per species versus sampling more species are being investigated by simulation elsewhere (Ahrens D., Krammer H.J., Fujisawa T., Fabrizi S., Vogler A.P., unpublished data). Population sizes for each species were assumed to be the same, and set in repeated simulations as 10^4^, 10^5^, 5×10^5^, and 10^6^ in turn. Larger population sizes increase the genetic variation within species and, consequently, are expected to make the transition from inter- to intra-specific processes harder to detect. Population sizes used here were chosen based on preliminary simulations to be representative of a trend from reciprocal monophyly of all species to the case in which few species are monophyletic on gene trees with population size of 5×10^5^ better reflecting this trend than just powers of 10. The range of population size covers the estimated effective population sizes of common eukaryotic species (Charlesworth 2009). Note that the performance of the GMYC method will depend on relative diversification and coalescent rates, not on absolute rates: for example, doubling population size and halving diversification rate would not change the shape of the resulting gene trees.
(C) Alternative diversification models: Simulation B assumes constant speciation rate and no extinction. However, this assumption is often violated in real data sets (Phillimore and Price 2008; Morlon et al. 2011; Etienne et al. 2012). We repeated the simulations with alternative diversification models. In C1, 30 species were sampled from a tree containing 50 species generated under a constant speciation rate. This model creates a recent deficit of speciation events, a situation that might arise due to incomplete species sampling or an actual slow down in the net diversification rate over time. Simulation C2 was simulated with constant speciation and extinction rate, with extinction rate 30% of the speciation rate. The model with extinction produces a recent excess of diversification events, the so-called “pull of the present” (Nee et al. 1994). Repeating the simulation with an extinction rate of 80% led to more extreme findings in the same direction as reported here. The GMYC model is designed to fit these different diversification processes by optimizing values of the scaling parameters as described earlier (i.e., *p*_spec_ < 1 for C1 and *p*_spec_ > 1 for C2). However, delimitation is expected to be easier in C1 than in simulation B, because terminal species divergence times will tend to be longer, and harder in C2, because terminal divergences will be shorter. Coalescent trees were simulated within species trees of both C1 and C2 with the same population and sample sizes as simulation B.
(D) Alternative population models: growing or declining populations: Simulation B assumes that species have had constant *N*_e_ over time. To check the ability of the scaling parameters to fit different population processes, we simulated two different models. In D1, we simulated gene trees assuming recent exponential growth within species. There are many potential versions that could be simulated, but we assumed that species were affected by an instantaneous population bottleneck *T*_b_ generations ago (arbitrarily choosing *T*_b_ to be half the age of the penultimate diversification event), followed by exponential growth to the present day. The rate of growth was assigned to generate a 10-fold increase in population size, and the severity of bottleneck was chosen so that the harmonic mean effective population size over the growth period was equal to *N*_e_, with the simulation repeated with *N*_e_ equal to 10^4^, 10^5^, 5×10^5^, and 10^6^ for comparability with simulation B. Effective population sizes before *T*_b_ generations ago were assigned as constant and equal to *N*_e_. In D2, we followed the same approach, but with an instantaneous growth in populations *T*_b_ generations ago followed by exponential decline. We predict that D1 should yield *p*_k_ < 1 and D2 should yield *p*_k_ > 1, reflecting a relative deficit and excess of recent coalescent events within species, respectively. We also predict that delimitation should be more accurate in D1 than in D2, because in D1 there will tend to be relatively shorter branching intervals near the most recent common ancestral node for a species. In reality, it is unlikely that all species would show the same population trends, but the simulations allow the broad effects of alternative models to be compared.
(E) Diversification with different sized populations: The above simulations assume that population sizes are equal among species. To investigate the effect of varying population sizes, we simulated effective population sizes drawn from a log-normal distribution with means equal 10^4^, 10^5^, 5×10^5^, and 10^6^ in successive simulations. We are unaware of any comparative data on effective population sizes among species, but species abundances typically follow a roughly log-normal distribution (Green and Plotkin 2007). Population sizes of each species of the species trees simulated in B were assigned from a log-normal distribution with means equal to 10^4^, 10^5^, 5×10^5^, and 10^6^ in successive simulations. Ancestral species were assigned the population size of the species with the lower index in the representation in Ms format.
(F) Alternative sampling scheme: random sample across the clades: Even if the alternative model is true, random sampling could bias the detection of true clusters if there is no prior knowledge of entities. To check the effect of random sampling, we simulated gene trees sampling 150 individuals in total, but choosing individuals at random with respect to species membership. Simulation F1 mirrored simulation B, with equal population sizes among species and hence equal probability that a sampled individual belongs to any species. In F2, we assumed log-normally distributed population sizes and species are sampled in proportion to their population sizes, that is, a species with twice the population size is likely to be sampled twice as often. Note that this simulation addresses the question of how the proportion of singletons within samples affects the accuracy of delimitation (Lim et al. 2011), since many species are sampled only once by chance.
(G) Geographical structure within species: For this scenario, we assumed that each species divided into two populations halfway along its terminal branch in the species tree and that two individuals were sampled from population 1 and three individuals from population 2. We then assigned a migration rate, *m*, such that *N*_e_*m* has a random uniform distribution between 0 and 1. All species in a given species tree were assumed to have the same migration rate, but a different migration rate was chosen for replicate species trees to explore how the performance of the GMYC method varied with migration rate. For *m*= 0, the two populations are completely isolated and the method should detect two separate clusters within each species. For higher *m*, the method should gradually shift from delimiting populations as clusters to delimiting the species.
(H) Sequence simulation and reconstructed gene trees: All simulations above assume that the true genealogy of sampled genes is known. In reality, however, true gene trees are unknown and estimated from DNA sequences. The uncertainty of tree inference may reduce the performance of delimitation. To assess the effect of tree inference, we conducted sequence simulations followed by tree inference and delimitation. DNA sequences were simulated along the branches of gene trees in simulation B using Seq-Gen (Rambaut and Grassly 1997), using the HKY+G model and average mutation rate of 0.02 per million generations (Brower 1994). Sequence lengths of samples were taken from a uniform distribution between 200 and 1800, which is the range of mitochondrial protein-coding genes (Luo et al. 2011). Gene trees were inferred from the simulated sequences using RAxML (Stamatakis 2006) with 100 bootstrap pseudoreplicates, then made ultrametric with the molecular clock assumption using the Langley–Fitch method implemented in r8s (Sanderson 2003). Identical sequences (haplotypes) were pruned to a single copy before implementation, because of known problems for GMYC caused by identical sequences (Monaghan et al. 2009): zero length terminal branches lead to calculation of an infinite coalescent *λ*.

Both the single-threshold and multiple-threshold algorithms were run on the simulated gene trees. The 95% confidence set was constructed for each simulation run first using all models defined by single thresholds and then adding models found with the multiple-threshold search. The size of the confidence set was recorded, and the rate of false negatives was measured as the rate of erroneously including the null model in the confidence set when the alternative model was true. When the null model was the true model, the rate of excluding the null model from the confidence set was calculated as the rate of false positives. The average proportion of species correctly delimited with an exact match was recorded as a measure of accuracy. Support values described above were calculated for each run of the simulations and their means were used to summarize the uncertainty of delimitation. ML estimates of numbers of clusters and scaling parameters for both speciation and coalescent part were also recorded. For simulation H, the GMYC results were compared with clusters inferred using a 2% distance threshold on a neighbor-joining tree of the simulated sequences.

To explore the effect of tree shape on delimitation, separately from the major differences among simulations, several tree-shape indices for both simulated species and gene trees were calculated. Mean branching times (Mean *T*_spec_) and *γ* statistics (Pybus and Harvey 2000) of the species trees were recorded to summarize the pattern of speciation events. The *γ*-statistic describes a tree's departure from the constant speciation model: *γ* < 0 indicates an apparent decrease of speciation rates, and *γ* > 0 indicates an apparent increase in speciation rate toward the tips. Note that the scaling parameters *p* correspond with *γ* as both indicate departure from constant speciation (e.g., when *γ* < 0, *p*< 1 is expected). Colless's index (*I*_colless_, Agapow and Purvis 2002) was calculated for each species tree to check the effect of tree imbalance on delimitation. Mean *Tmrca* (Mean *T_mrca_*) across species on gene trees was used as a measure of variation within populations. Multiple regression analysis was then performed with the number of exact matches as the response variable and the gene-tree indices and effective population size as explanatory variables. The importance of each index in predicting performance was expressed as its Akaike weight across models including it. For simulation G, the effect of the degree of population structure on delimitation success was also assessed with a regression analysis. All data processing and analyses were performed in R (R Development Core Team 2010) using the splits package (Ezard et al. 2009) with custom scripts, and the APE and apTreeshape packages (Paradis et al. 2004; Bortolussi et al. 2006). The latest version of GMYC in the splits package with the new formulation of parameters (version ≥1.0–15) is available at http://r-forge.r-project.org/projects/splits and in the Supplementary material deposited in Dryad. Note that R-forge only maintains versions compatible with the latest version of R. Previously available versions of GMYC used the previous formulation of threshold times.

### Case Studies: Tiger Beetles

Single-threshold versions of GMYC were applied to the data of Pons et al. (2006) and Pons et al. (2011). Solutions were compared to those obtained with the previous versions. Pons et al. (2006) sampled 468 individuals of tiger beetles of the genus *Rivacindela* from across 65 sites in Australia. The *Rivacindela* group was largely undescribed and, therefore, the samples were chosen from 108 local sets of individuals to represent their morphological and geographical diversity. Up to five individuals were sampled per morphospecies per site. Pons et al. (2006) sampled 161 individuals of the genus *Neocicindela* across New Zealand from 13 known taxa. Multiple individuals were sampled per site together with additional representatives of described species to cover their known range. Gene trees were inferred from three mitochondrial gene regions (cytochrome oxidase subunit 1, cytochrome *b* and 16S rRNA plus adjacent regions) in both studies. We used the dated gene trees from the source paper in both cases.

## Results

### Error Rates

**Figure 2:**
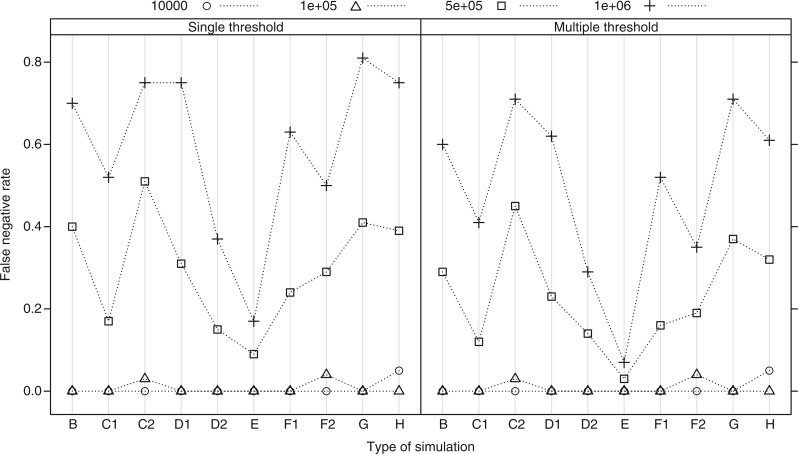
The mean false negative rate for each type of simulation and each effective population size from a sample of 100 simulated trees for each simulation type and population size combination. Simulation types are described in the ‘Methods' section: B is the constant speciation rate scenario; C1 is the incomplete sampling of species scenario; C2 is the constant background extinction rate scenario; D1 is growing populations; D2 is declining populations; E is variable population sizes among species; F1 is random sampling across the clade, equal population sizes; F2 is random sampling across the clade, unequal population sizes; G is with geographical structure within species; H is from inferred trees from simulated sequence data.

The rates of false positives (rejecting the null hypothesis at a 95% level when it is true) were *α* = 0.02 and *α* = 0.07 for the single- and multiple-threshold methods, respectively. The multiple-threshold method is, therefore, marginally less conservative than the single-threshold method. The rate of false negatives (accepting the null hypothesis when the alternative hypothesis is true) was zero or near zero in all simulations with low *N*_e_ (10^4^ and 10^5^, Fig. 2). Both methods, therefore, perform with similarly high power when effective population sizes are low relative to branching times between species. The rate of false negatives rose steeply, however, for high *N*_e_ (5×10^5^ and 10^6^, Fig. 2). The multiple-threshold model had greater power in all cases, for example, rejecting the null model in 1.1–2.4 times more cases than the single threshold when *N*_e_ = 10^6^.

Error rates also varied with the generating processes used in the simulations. As predicted, the rate of false negatives was always lower in C1 (decreasing apparent diversification rate = longer divergence times between closely related species) and higher in simulation C2 (increasing apparent diversification rate = shorter relative divergence times between closely related species) than in simulation B (constant diversification rate). Contrary to predictions, the rate of false negatives was lower in D2 (recently declining populations) than in either D1 (recently growing populations) or B. With log-normally distributed *N*_e_ (E), the rate of false negatives was much lower than the equivalent simulations with equal population sizes across species. Random sampling by clade rather than by species led to marginally reduced false negative rates when species had the same *N*_e_ (F1 vs. B, Fig. 2), but to increased false negative rates when species had different *N*_e_ (F2 vs. E, Fig. 2). Varying the degree of migration between two subpopulations within each species did not greatly affect the false negative rate (G, Fig. 2). The error rates of the reconstructed gene trees were comparable with the ones of the true gene trees except for *N*_e_ = 10^4^ (H, Fig. 2), in which case they were marginally higher.

### Accuracy of Delimitation

**Figure 3:**
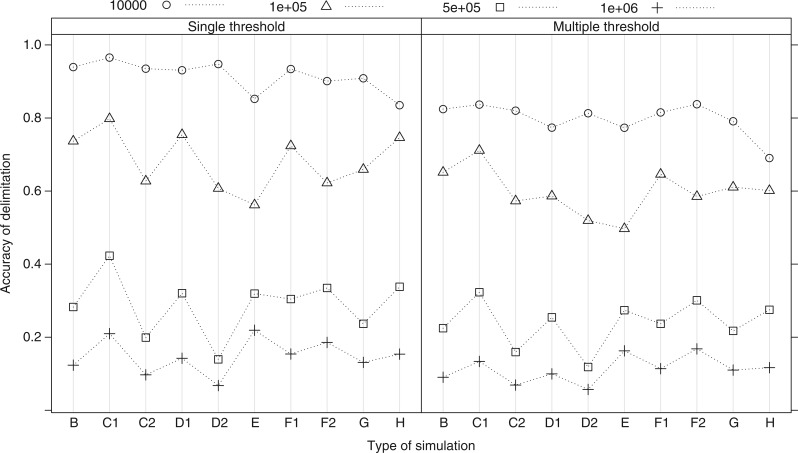
The mean accuracy of delimitation, expressed as the proportion of species correctly delimited, across the simulation types (B to H, see legend to Fig. 2) and across the four effective population sizes. Each point summarizes accuracy across 100 simulated trees.

The mean percentage of species that were delimited correctly with exact match, which we refer to as accuracy, fell from over 90% with *N*_e_ = 10^4^ to below 20% with *N*_e_ = 10^6^ (Fig. 3). The accuracy of delimitation decreased marginally with the multiple-threshold method in all cases (0.6–0.9 times less accurate than the single threshold). Among the different simulations, accuracy was higher in C1 (decreasing apparent diversification rate) than C2 (increasing apparent diversification rate) and in D1 (recently growing populations) than D2 (recently declining populations) at all *N*_e_, as predicted. The apparent discrepancy between results for error rates and accuracy in the relative performance of the methods in D1 and D2 is explained because the excess of recent coalescent events in D2 created artefactual clusters within species: the null model was more easily rejected, but only because of incorrect delimitation of clusters. Similarly, although simulation E (lognormal *N*_e_) displayed much lower false negative rates than simulation B, the accuracy did not differ greatly and indeed for higher *N*_e_ was lower than in simulation B (Fig. 3).

The accuracy of reconstruction was not greatly affected by random sampling across the clade rather than by species (B vs. F1 and F2, Fig. 3), indeed being marginally higher at low *N*_e_ even though a relatively high proportion of species were represented only by one or two individuals (simulation F1: number of samples per species ranged from 1 to 18, with 3.3% and 8.7% of species represented by 1 and 2 sequences, respectively; simulation F2 number of samples per species ranged from 1 to 67, with 18.2% and 15.3% of species represented by 1 and 2 sequences, respectively). Similarly, varying the degree of migration between two subpopulations within each species did not greatly affect the accuracy compared to the equivalent simulation assuming no structure within species (G vs. B, Fig. 3).

In simulation H, many sequences had to be pruned as identical haplotypes due to low variation. When *N*_e_ was 10^4^, 67% of sequences were removed and 49% of species were represented only by single sequences (singletons): this had the lowest accuracy of all simulation types but accuracy was still above 80% with the single-threshold method. The proportion of identical sequences and singletons decreased as *N*_e_ increased (singleton = 5% and identical = 33% for *N*_e_ = 10^5^. Singleton ≪ 0.01% and identical = 7% for *N*_e_ = 10^6^) and in those simulations accuracy was unaffected compared to analyses of the true genealogy (H vs. B, Fig. 3). Accuracy was, therefore, unaffected by inferring the genealogy from DNA sequences except when intraspecific variation was low enough to yield numerous identical sequences.

### Uncertainty

The size of the 95% confidence set of models increased as *N_e_* increased (Fig. S2). Trends among different simulations mirrored those for accuracy: simulations that displayed a higher accuracy retained a smaller confidence set. The distribution of GMYC support values also shifted similarly among simulations. For example, in simulation B with the single-threshold method, the mean support value across nodes was 0.96 for *N*_e_ = 10^4^, 0.63 for *N*_e_ = 10^5^, 0.35 for *N*_e_ = 5× 10^5^, and 0.30 for *N*_e_ = 10^6^. This demonstrates that the method of judging uncertainty adequately reflects the reduced performance of the model when the shift in branching patterns within versus between species is less distinct.

### Parameter Estimation

**Figure 4:**
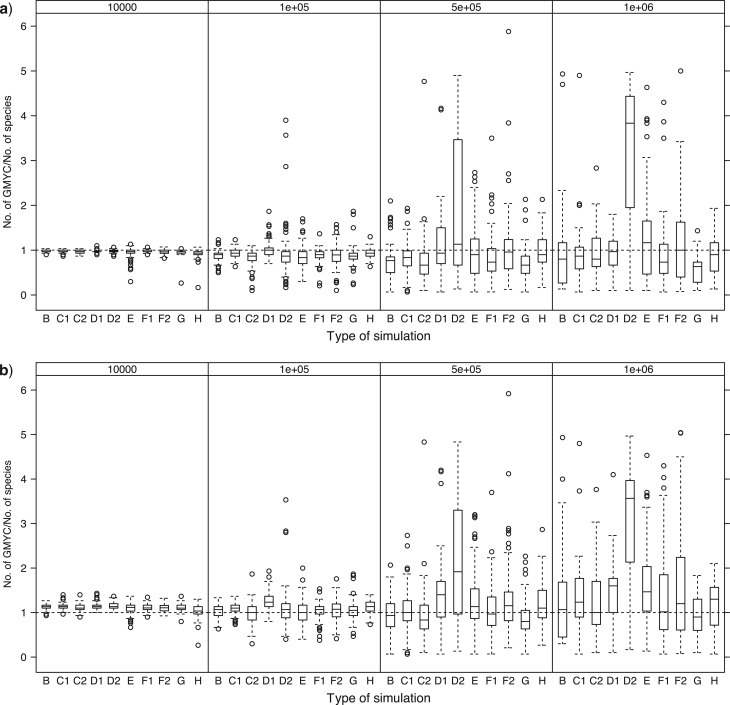
The estimated number of species per true species as recovered by a) the single-threshold and b) multiple-threshold versions of GMYC. Results are shown for each combination of simulation type (B to H) and effective population size. The line within each box shows the median, the box limits show the inter-quartile range, and whiskers/points indicate extreme values.

The estimated number of clusters, that is, inferred species, matched well with real number of species for *N*_e_ = 10^4^ across all simulations (Fig. 4). Estimates declined and their range increased with increasing population size and the differences among the simulation types matched those reported for accuracy above. The discrepancy was largest in simulation D2 because of the spurious clusters within species discussed above. The multiple-threshold method tended to overestimate the number of clusters marginally compared to the single-threshold method, even when *N*_e_ was small. Variation among the simulation types mirrored those reported for accuracy above, with higher accuracy associated with better estimates of the number of clusters. The tree inference did not affect the number of estimated clusters even with the amounts of singletons for *N*_e_ = 10^4^ (Fig. 4, H).

**Figure 5:**
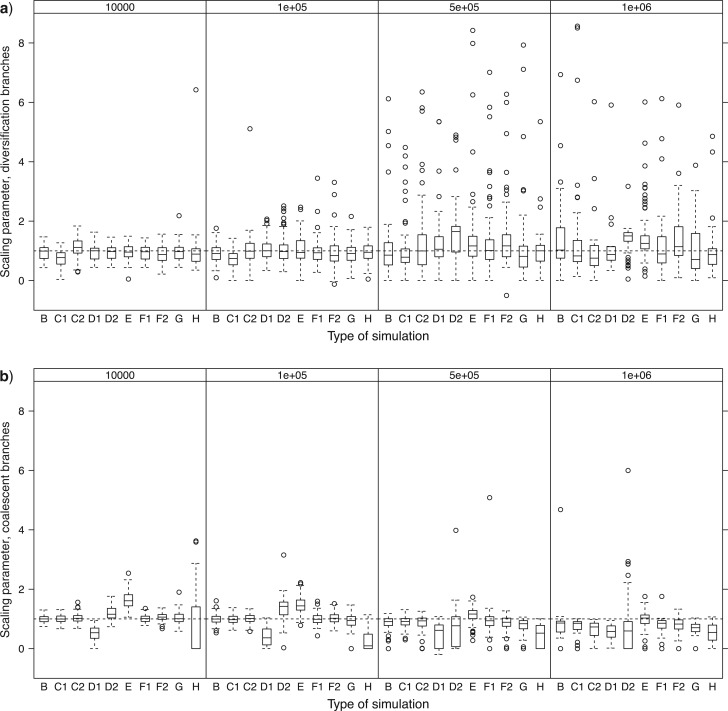
The estimated scaling parameters for a) the diversification branching process and b) the coalescence branching process across each combination of simulation type (B to H) and effective population size, using the single-threshold method. The horizontal dashed line indicates a value of 1, which is expected under the default model assumed in simulation B of a constant-rate diversification process and a neutral coalescent process with no substructure and constant population sizes. The multiple-threshold results are in Fig. S3 and follow similar patterns across simulations.

Estimated scaling parameters correctly inferred the different diversification processes in simulations C1 and C2 when *N*_e_ was low (Fig. 5a). In C1, estimated values were smaller than 1 (median = 0.77, *V* = 377, *P* ≪ 0.001 for *N*_e_ = 10^4^ Wilcoxon test), which matches a recent deficit of diversification events. The estimates for C2 were larger than 1 (median = 1.12, *V* = 3404, *P* = 0.003 for *N*_e_ = 10^4^ Wilcoxon test), which matches a recent excess of diversification. For higher *N*_e_, the estimates deviated from the expected patterns and no longer reflect the patterns of diversification process (median = 1.16, *V* = 3509, *P* < 0.001 for C1, median = 1.47, *V* = 3900, *P* ≪ 0.001 for C2, *N*_e_ = 10^6^ Wilcoxon test): for example, in simulation C, incorrectly assigning recent speciation events as coalescence events (shown by the underestimate of number of clusters in C2 and *N*_e_ = 10^6^, Fig. 4) meant that the model no longer optimized a relative excess of recent diversification events. The estimates of scaling parameters for the coalescent process also matched with the simulated population processes, being smaller than 1 in D1 and larger than 1 in D2 (Fig. 5b, median = 0.53, *V* = 0, *P* ≪ 0.001 for D1 and median = 1.16, *V* = 4579, *P* ≪ 0.01 for D2, *N*_e_ = 10^4^ Wilcoxon test). These estimates were also affected by increased *N*_e_ and resulted in values lower than 1 for all simulations at the highest *N*_e_ (median = 0.77, *V* = 0, *P* ≪ 0.001 for D1 and median = 0.82, *V* = 782, *P* ≪ 0.001 for D2, *N*_e_ = 10^6^ Wilcoxon test).

### Population Structure

**Figure 6:**
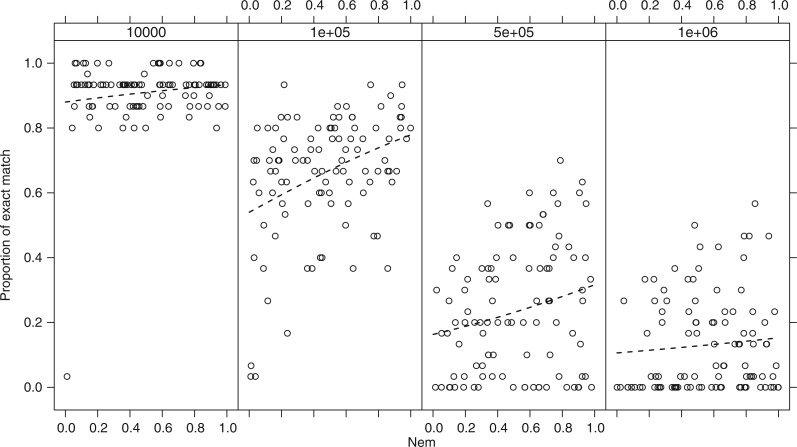
The accuracy of delimitation, represented as the proportion of species that were correctly delimited, across simulations varying in the level of migration between two subpopulations (*m*). Separate plots are shown for each of the four effective population sizes used in the simulations.

Varying the degree of migration between two subpopulations within each species did not greatly affect the accuracy of delimitation or any other metrics of the performance of the methods when averaged across all simulations (G, Figs. 2–5). Although marginal declines of accuracy were observed in intermediate *N*_e_ values (Fig. 6, 10^5^ and 5×10^5^), there were no significant effects of migration and its interaction with *N*_e_ on the accuracy (*z*= 1.16, *P* = 0.24 for *N*_e_*m* and *z*= 0.27, *P* = 0.79 for the interaction of *N*_e_ and *N*_e_*m*, GLM with binomial error).

### Reconstructed Gene Trees and Comparison with the Distance-based Method

The length of sequence did not have significant effect on accuracy when all effective population sizes were considered in the regression modeling (*z*= 0.75, *P* = 0.46, GLM with binomial error Fig. S4). Increasing accuracy with sequence length only occurred weakly when *N*_e_ = 10^4^, and there was no significant interaction between *N*_e_ and sequence length. The GMYC method generally outperformed distance-based clustering with 2% cut-off (relative accuracy of 2% method vs. GMYC: 72% vs. 84% when *N*_e_ = 10^4^, 77% vs. 75% when *N*_e_ = 10^5^, 26% vs. 34% when *N*_e_ = 5×10^5^, and 7% vs. 15% when *N*_e_ = 10^6^). The GLM showed that the overall accuracy was significantly lower for the 2% distance threshold method (*z*= -2.22, *P* = 0.026).

### General Tree Shape Metrics and Their Effects on Accuracy of Delimitation

The average *γ*-statistic was -0.016±0.033 (min = -3.05, max = 3.14), whereas the average Colless imbalance index (*I*_colless_) was 71 ± 0.58 (min = 28,max = 143). Mean branching time (Mean *T*_spec_) and mean coalescent time (Mean *T*_coal_) ranged from 0.13 to 0.49 and from 0.0005 to 0.25 with average 0.31±0.002 and 0.06±0.007, respectively. Effective population size (*N*_e_), *γ* statistics, and mean coalescent time had the greatest effect on accuracy (Table 1). They were included in all models within the 95% confidence set using the single threshold (relative importance = 1.0) and the estimates of their slopes were significantly different from 0: for each metric, accuracy increased as its value decreased. Mean *T*_coal_ was not significant with the multiple thresholds. Mean branching time had low relative importance (0.29) and its estimate was not significant. The tree imbalance index was of lowest relative importance in both treatments, as would be expected because the method relies on internode branching intervals rather than topology.

**Table 1.** The relative importance (Σ*w_i_*) of tree shape variables in explaining the accuracy of delimitation across simulations, and their model averaged coefficients 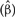 plus standard errors (SE)

Method	Single		Multiple	
	Σ*w_i_*	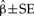	Σ*w_i_*	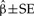
*N*_e_ ^a^	**1**	−**2.78×10^−6^**	**1**	−**3.09×10^−6^**
		**3.15×10^−7^**		**3.09×10^−7^**
*γ*^b^	**1**	−**0.42**	**1**	−**0.23**
		**5.95×10^−2^**		**6.30×10^−2^**
*I*_colless_^c^	0.26	7.63×10^−4^	0.21	3.36×10^−4^
		3.16×10^−3^		2.50×10^−3^
Mean *T*_spec_^d^	0.29	−1.29×10^−3^	0.33	5.21×10^−3^
		1.33×10^−2^		1.57 ×10^−2^
Mean *T*_coal_^e^	**1**	−**8.6**	0.76	−2.88
		±**2.33**		±2.37

^a^Mean effective population size, variables with a coefficient significantly different from zero are highlighted in bold.

^b^Gamma statistics of Pybus and Harvey (2000).

^c^Colless's tree imbalance statistic.

^d^Mean divergence time between species.

^e^Mean coalescence time across species.

### Tiger Beetles

The two data sets of tiger beetles differed in the degree of uncertainty in delimitation. The number of delimited species (numbers of clusters and singletons) ranged from 40 to 51 (ML estimate 48) with 95% confidence in *Rivacindela* (Fig. S5). Note that the confidence limits are wider than the previously reported confidence limits (47–51) based on our former method of reporting solutions within two log likelihood units of the ML solution. The 95% confidence set included 12 of the 467 delimitation models evaluated and 27 of the 43 nodes chosen to delimit species in the best model had GMYC support values of 1.0 (mean GMYC support of nodes included in the 95% confidence set = 0.76). In contrast, the method delimited between 17 and 55 species (ML estimate 45) in *Neocicindela* at the 95% confidence level (Fig. S6). Again, the confidence interval was broader than previously obtained (32–51, Pons et al. 2011). The 95% confidence set included 31 of 160 models compared and no nodes had GMYC support values of 1.0 (mean GMYC support of nodes included in the 95% confidence set = 0.33). The reasons for the differences in the strength of clustering in the two clades have been discussed elsewhere, but could include either the lower sample of individuals in *Neocicindela* or the stronger geographical structuring of *Rivacindela* species on fragmented salt lakes (Pons et al. 2011).

## Discussion

The GMYC method for delimiting clusters from single-locus gene trees has been widely used to delimit putative species from DNA barcode-type data, yet its performance has not previously been tested extensively on simulated data (but see Papadopoulou et al. 2008; Lohse 2009; Esselstyn et al. 2012 for simulations of particular features). Our analyses show that both the single- and multiple-threshold versions are close enough to the target error of 0.05 to be suitably conservative when applied to samples that might indeed derive from a single, unstructured species cluster. For applications that require a test of this null hypothesis, we recommend either simulating trees under the null model to calculate an exact *P*-value or a chi-square test comparing the single-threshold GMYC model to the null model for a conservative *P*-value.

Our simulations also confirm previous intuition about the key parameters determining the power and accuracy of GMYC. When effective population sizes were low relative to species divergence times, then species were monophyletic and the method delimited clusters accurately, irrespective of detailed assumptions concerning the diversification and population processes. As the mean or variance in effective population size increased, then the accuracy of delimitation declined. In the extreme case, species were no longer reciprocally monophyletic and therefore could not be accurately delimited by the method (which assumes reciprocal monophyly). These results match with the findings of Esselstyn et al. (2012) who compared species richness estimates for trees simulated with different ratios of *N*_e_ to speciation rate.

Accuracy varied among the different simulations mostly in accordance with predicted patterns based on the effects of particular processes. The scaling parameters did accurately represent the diversification and coalescent processes in the simulations when population sizes were low. Nonetheless, incorporating more realistic macroevolutionary models might improve the performance with real data. For example, empirical studies have reported that the background extinction rate can be around 90% of speciation rate (Ricklefs 2007; Etienne et al. 2012). This will tend to blur the sharpness of transition from interspecific to intraspecific branching, and therefore to reduce the accuracy of the GMYC method. Although the scaling parameter was able to fit this trend in apparent diversification rate, more accurate delimitation might be obtained by incorporating extinction explicitly in the model. A final assumption concerning the diversification part of the model is that diversification rates are uniform across all clades. Again, models allowing for this departure could be implemented (Morlon et al. 2011).

The performance of the method also depends on sampling in several ways. Increased sampling of individuals per species will increase performance. Here, we only simulated a sample of five individuals per species, to reflect the likely limited sampling within species for broad biodiversity surveys. Increasing that number might increase the apparent branching rate within clusters and, hence, the ability of the method to detect a threshold in branching rates (see Reid and Carstens 2012). For example, in the tiger beetle studies, the mean number of individuals per delimited species was 9.75 (min = 1,max = 63, 10% and 13% of clusters represented by 1 and 2 sequences, respectively) for *Rivacindela*, in which high support values were obtained, but only 3.58 (min = 1,max = 21, 51% and 9% of clusters represented by 1 and 2 sequences, respectively) for *Neocicindela*, in which GMYC support was much lower. Conversely, increased sampling of species within a clade (but holding the number of individuals sampled per species constant) might reduce the ability to delimit species included in the sample. This occurs because random omission of species reduces the probability that each species' closest related species will be sampled, and hence reduces the chance of finding nonmonophyletic species. This result has been shown empirically for increasing spatial scales of sampling in European water beetles (Bergsten et al. 2012). The effects of numbers of individuals per species versus number of species sampled on the accuracy of the method are being investigated by simulation elsewhere: GMYC can work accurately with as few as 3 species, but it is more robust with more than 10 species (Ahrens D., Krammer H.J., Fujisawa T., Fabrizi S., Vogler A.P., unpublished data).

Finally, although random sampling across the clade versus random sampling within each species might be expected to affect the method, our results showed that the effect was small relative to the effects of *N*_e_ (compare B and F2 in Fig. 3). The proportion of species represented by only one or two sequences in these simulations (12% in F1 and 33.5% in F2) reflects realistic levels of rarity in many biodiversity surveys (Lim et al. 2011). This result means that the method is likely to be little affected by whether samples are taken evenly based on prior inferences on species limits (as in many DNA barcoding studies of multicellular eukaryotes) or without such knowledge (as in studies of unculturable bacteria or eukaryotes from environmental samples). Our simulation code is available in the Supplementary material for users wishing to investigate possible effects of their own sampling design.

We found that relaxing the constraint of the single threshold does not lead to increased performance of the method. The multiple-threshold version yields fairly similar results to the single-threshold version, but with a tendency to over-split. Where this happens, the node recovered by the single-threshold version is normally still included in the 95% confidence set of MRCA nodes, but with reduced confidence. While it is possible that future work could describe a multiple-threshold algorithm that could improve delimitation, for example, more exhaustive searching using Bayesian Markov Chain Monte Carlo (Reid and Carstens 2012), we believe instead that this might reflect a fundamental limitation to the potential for the method. Imagine an extreme case in which each species had a different time to MRCA and rate of branching within clusters. In this case, there would be no average signal across the tree from which to reconstruct the shift in branching process. It is only when the shifts do coincide at a particular level in the tree that we are able to find statistical evidence for clustering. Our results do not rule out that the multiple-threshold version might yield improved results in some cases (which would be signified by the exclusion of an MRCA node detected from the single-threshold version). We recommend therefore that the multiple-threshold version be used, with caution, as a way to explore how delimitation changes when the assumptions of the single threshold are relaxed.

The GMYC model has previously been criticized for assuming that species comprise a single, unstructured population. Geographical structure is a common feature of many real species and, combined with nonrandom sampling of species ranges, this could introduce biases into species delimitation. Previous simulations to investigate this problem concerned the ability of GMYC to delimit clusters along a continuum from a single population to a population structured into subpopulations with no gene flow (e.g., Papadopoulou et al. 2008, 2009; Lohse 2009). In such cases, it was shown that GMYC could delimit partially isolated populations as separate species, especially if linking populations were not sampled. More pertinent for most real applications of GMYC, however, is to consider how geographic structure within each species affects the ability of GMYC to delimit species across a wider clade comprising multiple distinct species. Our simulations here show that, as long as the conditions for accurate delimitation of species are met (i.e., low effective population size relative to species divergences), then further geographic structuring within species does not greatly affect the accuracy of the method: because of the low variation within each species, the threshold was optimized at the correct level of the tree.

The GMYC method requires identical sequences to be removed beforehand because zero length terminal branches hampers the likelihood estimation; that is, the Moran estimator of *λ* cannot be properly evaluated due to zero total branch length within clusters. This can be particularly problematic when subject gene trees are reconstructed from slowly evolving markers, which leads to reduced total sample size and variation within species. However, the simulation studies showed that the method is tolerant to moderate amount of identical sequences and singletons, delimiting them by correctly assigning them speciation branching. The accuracy on reconstructed gene trees—simulated for a rate of variability and range of marker lengths typical of mtDNA and other single-locus studies—was also comparable to the results using the true genealogy. In addition, the GMYC generally performed better than the 2% cut-off clustering, indicating the advantage of varying the boundary of species over the fixed cut-off value, although accuracy was equivalent when *N*_e_ = 10^5^. The relative performance of these methods will depend on typical levels of *N*_e_ and variation in substitution rates: in a recent survey of animal taxa, GMYC and use of a 3% threshold yielded similar results (Tang et al. 2012).

One clear limitation of GMYC is that it assumes species are monophyletic. This assumption is violated for real species when divergence time was insufficient for entities to gain monophyly (Hudson and Coyne 2002). The decline of the matches between true entities and estimated clusters reflects this violation. According to theoretical studies, the time for reciprocal monophyly of a neutral marker to arise with 95% probability between a pair of species is 8.7 Ne for a single nuclear marker and 2.2 Ne for mtDNA (Hudson and Coyne 2002). The range of nonmonophyly of species in our simulation studies were between 0% and 87% (median 20%), which covered well the rate of 23% reported from a meta-analysis of population genetic studies (Funk and Omland 2003). The proportion of non-monophyletic species was strongly correlated with the accuracy of delimitation (Fig. S7). The predicted accuracy on 20% of non-monophyly was around 50% of exact matches.

Signatures of recent speciation events, therefore, cannot be detected with the method (or any other method using monophyly of single genes), although 50% of accuracy could be still useful in the rapid assessment of species diversity. This limitation can be addressed with multilocus analyses, which permit delimitation of more recently diverged sister species (Knowles and Carstens 2007). However, the methods currently require prior hypotheses of species populations and would be computationally intensive for large sample sizes. A key challenge for multilocus approaches is to find efficient algorithms for searching alternative delimitations (Yang and Rannala 2010). Note that GMYC can be used to delimit clusters from any ultrametric trees, including those derived from concatenated multilocus data: although it will be less powerful than multilocus methods in sexual organisms (Knowles and Carstens 2007), perhaps it could be used to find starting delimitations for evaluation with multilocus approaches.

## Conclusions

The GMYC method was devised originally as a method for delimiting species from single-locus gene trees in the absence of any additional information. It does so by detecting genetic clustering beyond levels expected in a null model that all sampled individuals belong to a single interacting population. In groups in which effective population sizes tend to be low and divergence times between species tend to be high, then the method is accurate and conservative. In many eukaryotes and prokaryotes, there are strong patterns of genetic clustering apparent in gene trees for clades or environmental samples, indicating that the conditions for GMYC are often met. Wherever additional information is available, such as the sampling locality of individuals, quantitative or qualitative information on phenotypic traits, or additional gene sequences, then this information should be used to cross-check the entities delimited by GMYC. In larger eukaryotes, such information is usually available, and the utility of GMYC comes from providing an objective means to delimit genetic clusters for comparison with other data. Efforts should be made to sample individuals across a representative range occupied by the species and to keep in mind potential limits of the accuracy of the method discussed above. The task that GMYC is most well suited to is delimiting units of diversity when only DNA sequence data are available, such as in environmental DNA surveys. There it provides an evolutionary framework to complement existing pragmatic methods based on empirically based thresholds.

## Supplementary Material

Supplementary Data

## Supplementary Material

Data files and/or other supplementary information related to this paper have been deposited at Dryad under doi: 10.5061/dryad.0hv88.
