# Supplementary material for: Delimiting Species Using Single-Locus Data and the Generalized Mixed Yule Coalescent Approach: A Revised Method and Evaluation on Simulated Data Sets
Source: Syst Biol. 2013 Jun 14;62(5):707–24. doi: 10.1093/sysbio/syt033 (PMC3739884; doi:10.1093/sysbio/syt033)
Supplement: Supplementary Data [file supp_62_5_707__index.html]

Delimiting Species Using Single-Locus Data and the Generalized Mixed Yule Coalescent Approach: A Revised Method and Evaluation on Simulated Data Sets — Delimiting Species Using Single-Locus Data and the Generalized Mixed Yule Coalescent Approach: A Revised Method and Evaluation on Simulated Data Sets — Supplementary Data 

# Delimiting Species Using Single-Locus Data and the Generalized Mixed Yule Coalescent Approach: A Revised Method and Evaluation on Simulated Data Sets

## Supplementary Data

files

**Files in this Data Supplement:**

- Supplementary Data - docx file
