# Supplementary material for: Delimiting Species Using Single-Locus Data and the Generalized Mixed Yule Coalescent Approach: A Revised Method and Evaluation on Simulated Data Sets
Source: Syst Biol. 2013 Jun 14;62(5):707–24. doi: 10.1093/sysbio/syt033 (PMC3739884; doi:10.1093/sysbio/syt033)
Supplement: Supplementary Data [file supp_syt033_Fujisawa.Appendix.docx]

APPENDIX: Computational complexity of the search of species defining nodes

The number of combinations of species defining MRCAs with a tree with *n* tips, F_n_, is defined as follows. When the number of tips is 2, F_2_ is obviously 2; either splitting the root into tips or keeping the root as an MRCA (Fig. S3.1a). F_n_ for tips more than 3 can be defined recursively. For example, with trees with 4 tips, F_4_ is 5 if the tree is balanced (Fig. S3.1b). The values of F for subtrees with 2 tips are F_2_=2, and possible combinations of MRCAs for the two subtrees are 2*2. The root node adds 1 to get F_4_=5. In general, F_n_ are represented by a product of two Fs from subtrees plus 1 (Fig. S3.1c). It is difficult to define F_n_ for a tree with 1 tip. However, F_1_ can be defined as F_1_=1 so that it does not break the recursive patterns. For example, F_3_=F_2_*F_1_ +1 = 3.


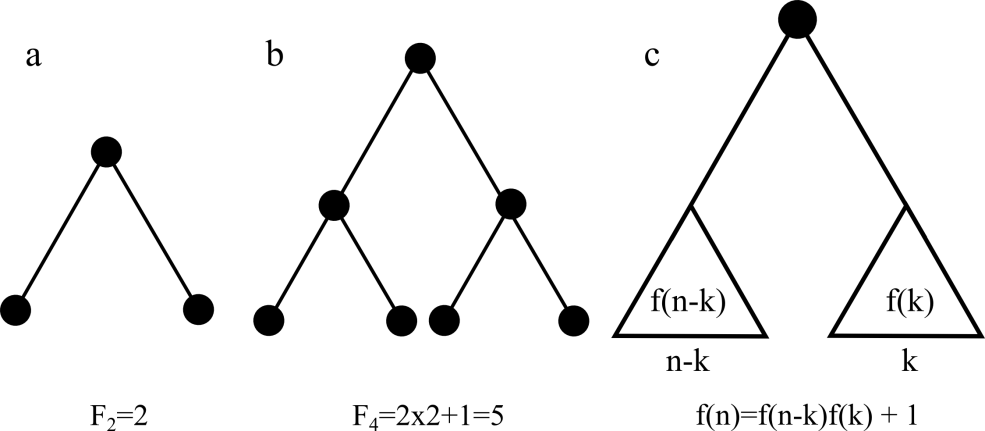


Figure S.3.1 Relations of number of tips, n, and F_n._

Therefore, the number of combinations of species defining nodes on a tree with *n* tips is given with the following recurrence relation;

$$\left\{ \begin{aligned} f\left( 1 \right)=1 \\ f\left( n \right)=f\left( n-k \right)f\left( k \right)+1 \end{aligned} \right. n\geq1, 1\leq k\leq n-1 (eq. S.1)$$

*f*(*n*-*k*) and *f*(*k*) denote the number of combinations on the left and right subtrees. Although it is impossible to obtain the exact closed form of *f*(*n*), the upper and lower bounds of *f*(*n*) can be analyzed.

## The lower bound of f(n): the best case

When k is always equal to 1, that is, a given phylogeny is completely imbalanced, the recurrence (eq. S.1) is reduced to,

$$f\left( n \right)=f\left( 1 \right)f\left( n-1 \right)+1$$

Since *f*(1) = 1,

$$f\left( n \right)=f\left( n-1 \right)+1 (eq.S.2)$$

The closed form of (eq.S.2) is *f*(*n*) = *n*. Then, F_n_ increases linearly with the number of tips for a given completely imbalanced tree.

## The upper bound of f(n): the worst case

For a given completely balanced tree, where the number of tips of subtrees is exactly the half of the original tree, the recurrence (eq.S.1) is,

$$f\left( n \right)={f\left( n/2 \right)}^{2}+1$$

This relation only holds when n=2*^l^*. Therefore,

$$f\left( 2^{l} \right)= {f(2^{l-1})}^{2}+1, l\geq1 (eq.S.3)$$

This recurrence generates the sequence 1, 2, 5, 26, 677, 458330,… and appears to be the worst case of the increase of F_n_. This sequence is the sequence A003095 in the On-Line Encyclopedia of Integer Sequences (OEIS foundation Inc. 2011), and the closed form of (eq.S.3) was obtained by Aho & Sloane (1973, equation 22).

$$f\left( n \right)= \left\lfloor k^{n} \right\rfloor, n=2^{l}, l\geq0$$

where, $\left\lfloor a \right\rfloor$ is the greatest integer less than or equal to *a* (i.e. floor of *a*) and,

$$k=f\left( 1 \right)exp\left( \sum_{i=0}^{\infty} 2^{-i-1}ln\left( 1+\frac{1}{{f(i)}^{2}} \right) \right)$$

k is numerically approximated in Aho & Sloane (1973).

$$k=\exp\left( \frac{1}{2}\ln\left( 2 \right)+ \frac{1}{4}\ln\left( \frac{5}{4} \right)+\frac{1}{8}\ln\left( \frac{26}{25} \right)+\ldots\right) \cong1.502837\ldots$$

Hence, in the worst case with a completely balanced tree, the number of possible combination of MRCAs is approximately $\left\lfloor{1.502}^{n} \right\rfloor$ with *n* tips. When n=128, this value is ${1.502}^{128}=4.11\times{10}^{22}$. The number of possible sets of species defining MRCAs, F_n_, grows exponentially with increasing tree size. F_n_ value ranges from the upper limit $\left\lfloor{1.502}^{n} \right\rfloor$ and the lower limit *n*, depending on tree balance. The exponential increase makes exhaustive search of the best set of MRCA nodes impractical. The single threshold method can effectively reduce the number of models down to the lower bound of F_n_, that is, *n* for *n* tips, while the computational time for the multiple threshold method expected to increase exponentially as its search space is likely to be proportional to the size of F_n_.

REFERENCES:

Aho, A.V. & Sloane, N.J.A. (1973) Some doubly exponential sequences. *Fibonacci Quarterly*, 11(4), 429-437.

OEIS foundation Inc., (2011) The On-Line Encyclopedia of Integer Sequences. http://oeis.org
